# Evaluation of Dried Blood Spot Sampling for Clinical Metabolomics: Effects of Different Papers and Sample Storage Stability

**DOI:** 10.3390/metabo9110277

**Published:** 2019-11-12

**Authors:** Oxana P. Trifonova, Dmitri L. Maslov, Elena E. Balashova, Petr G. Lokhov

**Affiliations:** Institute of Biomedical Chemistry, 10 building 8, Pogodinskaya street, 119121 Moscow, Russia; dlmaslov@mail.ru (D.L.M.); balashlen@mail.ru (E.E.B.); lokhovpg@rambler.ru (P.G.L.)

**Keywords:** postgenome medicine, mass spectrometry, dried blood spot, metabolomics, metabolite profiling

## Abstract

The dried blood spot (DBS) sampling has a lot of advantages in comparison with the “standard” venous blood collecting, such as small collection volume, painless and easy sample collection with minimal training required, stable and transportable at ambient temperatures, etc. The aim of this study was to determine the comparability of four different types of DBS sampling (HemaSpot™-HF Blood Collection Device, Whatman^®^ 903 Protein Saver Snap Apart Card, card ImmunoHealth™, and glass fiber strip ImmunoHealth™) for analysis of the global metabolites profile. All the samples were collected from the same person at the same time and stored at room temperature for four weeks in order to exclude all possible deviations deriving from biological variances and to evaluate sample storage stability. Metabolome profiling by direct injection of a deproteinized capillary blood DBS sample into an electrospray ion source of a hybrid quadrupole time-of-flight mass spectrometer was used. Differences in the metabolomics profile were found between the different DBS collection materials, especially for ImmunoHealth™ card and ImmunoHealth™ glass fiber strip. However, our results indicate that the analytical performance of all tested DBS sampling materials showed consistent results overall detected metabolites and no dramatic changes between them in the metabolic composition during the storage time.

## 1. Introduction

Blood is one of the most used biological fluids for research, diagnostic, health, and drug monitoring. Following the trend of development of precision medicine with emphasis on personalized approach and population health studies, tools that facilitate sample collection and enable patients to contribute to the expanding bioanalytical empowerment for health care are necessary. The dried blood spot (DBS) sampling has a lot of advantages in comparison with the “standard” venous blood collection [1,2]. Firstly, DBS sample collection is minimally invasive and therefore painless and can easily be performed by patients themselves. Usually, DBS samples are obtained by direct spotting from finger or heel pricks. Secondly, a small collection volume is required for DBS: less than 100 μL of capillary blood is needed to spot onto sampling paper compared to a minimum of 0.5 mL of blood in venous sampling. And finally, DBS samples are stable at ambient temperatures and offer possibilities for easy shipment and storage without the expense and infrastructure associated with maintenance of specific temperature (e.g., no requirement for freezing or dry ice using). Using this methodology, blood samples can be easily collected by the patient itself or his guardian with minimum training and sent by mail to the assigned laboratory. Clinical trials or cohort studies requiring collection, shipment, storage, and analysis of thousands of samples could benefit from these advantages. Several population-based studies employing the DBS samples and especially using self-sampling by study participants have been already reported [3,4,5].

DBS sampling has a long history and has shown its robust capabilities for newborn screening [6,7] and screening for a number of viral diseases, including HIV and viral hepatitis [8,9]. Today the analysis of DBS has been extended and has become a practice tool for many different applications ranging from therapeutic drug monitoring [10,11], pharmacokinetics [12,13], genomics [14], proteomics [15,16], to metabolomics [17]. Non-targeted and targeted metabolomics analyses have been performed using samples captured via DBS [18,19,20]. Until recently, the only cellulose-based paper cards have been available for DBS sample collection and just in the last several years a number of new DBS devices like strips, sticks, pens, and tips have been developed [21,22,23,24]. The main disadvantages of DBS are hematocrit effect and a variety of existing commercial DBS matrixes, which have not been yet validated or received regulatory approval for this method of sample collection, transport, and further clinical analysis [25,26,27].

The aim of this study was to determine the comparability of four different types of DBS sampling, including HemaSpot™-HF Blood Collection Device, Whatman^®^ 903 Protein Saver Snap Apart Card, card ImmunoHealth™, and glass fiber strip ImmunoHealth™ for analysis of the global metabolites profile. It should be noted that all of these DBS samples collection methods are permitted for research use only. Only the Whatman^®^ 903 paper is registered with the U.S. Food and Drug Administration (FDA) as an in vitro Class II medical device and used in most newborn screening programs throughout the world (Product Code - PJC). Therefore, currently Whatman 903 (W-903) is the only filter paper that has been widely used; for example, for viral diseases testing and therapeutic drug monitoring [28,29,30]. The HemaSpot™-HF Blood Collection Device and the glass fiber strip ImmunoHealth™ have been used in veterinary studies, such as to test blood samples collected from dogs for antibodies to Leishmania infantum [21] and strip-dried whole milk sampling technique for progesterone detection in cows by ELISA analysis of progesterone in cow milk [31], respectively. Here, we evaluated the effect of the four studied DBS sampling on analytes recovery and molecular stability of the DBS samples during storage at room temperature for metabolome profiling.

## 2. Results

### 2.1. Direct Injection-Based Metabolomics Analysis

**Figure 1:**
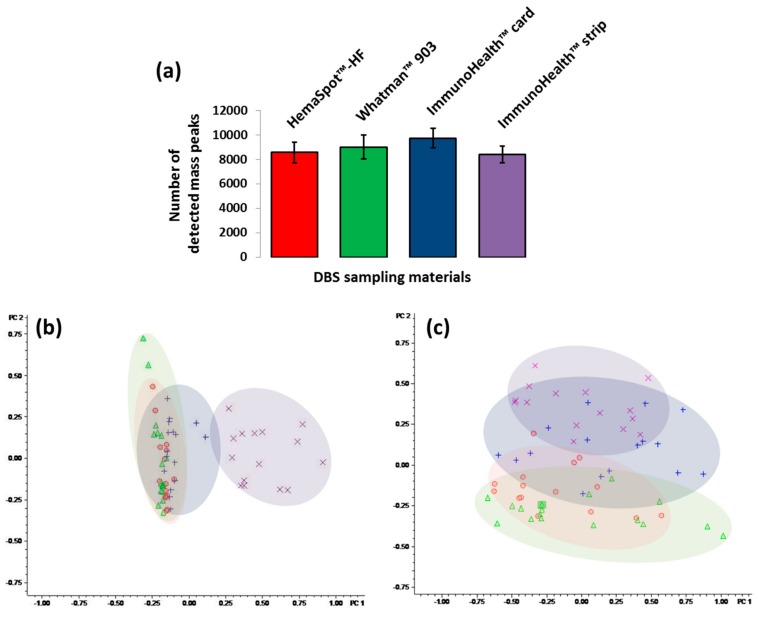
Number of detected mass peaks (mean ± SD) (**a**) and principal component analysis (PCA) of the mass spectrometry-based metabolomics data detected from the different DBS sampling: HemaSpot™-HF Blood Collection Device (o), Whatman^®^ 903 Protein Saver Snap Apart Card (∆), card ImmunoHealth™ (+), and glass fiber strip ImmunoHealth™ (×) before (**b**) and after (**c**) specific contaminant subtraction. Color code: red represents samples extracted from HemaSpot™-HF Blood Collection Device, green represents samples extracted from Whatman^®^ 903 Protein Saver Snap Apart Card, blue represents samples extracted card ImmunoHealth™, and violet represents samples extracted from glass fiber strip ImmunoHealth™.

To evaluate the best DBS sampling for metabolomics application, we analyzed the metabolomes extracted from HemaSpot™-HF Blood Collection Device, Whatman^®^ 903 Protein Saver Snap Apart Card, card ImmunoHealth™, and glass fiber strip ImmunoHealth™ by using a direct injection mass spectrometry (DIMS) method as a typical workflow designed for rapid untargeted analysis of the polar blood metabolome. The samples were analyzed in random order in five technical replicates to monitor variability in the number of detected mass peaks. Differences in the metabolomics profile were found between the different DBS collection materials, especially for card ImmunoHealth™ and glass fiber strip ImmunoHealth™. The highest number of mass peaks was detected in samples extracted from card ImmunoHealth™ (Figure 1a). In contrast, the least effective in terms of the number of detected mass peaks was glass fiber strip ImmunoHealth™. However, the difference between the average numbers of mass peaks detected in samples extracted from all four studied DBS sampling materials proved to be not significant. 

The PCA showed that samples extracted from glass fiber strip ImmunoHealth™ clustered separately from the other samples (Figure 1b). The first two principal components accounted for 70% of the total variance (PC1 – for 45% and PC2 – for 25%) and could clearly separate the metabolome extracts from the strip from the other samples. All other extracts clustered very closely, suggesting that from HemaSpot™-HF cartridge and cards Whatman^®^ 903 and ImmunoHealth™, a similar metabolome has been recovered. Differential metabolites detection specific to each DBS sampling (less than 15% of the detected features) due to interfering contaminants originating from the paper cards and glass fiber strip was observed. After subtracting a number of mass peaks related to the already known contaminants (polyethylene glycol, polypropylene glycol, etc.) [32,33] presented in high amounts, especially in samples extracted from ImmunoHealth™ card and strip the PCA couldn’t clearly separate the metabolome extracted from the studied DBS samplings (Figure 1c). Thus, it is fully confirmed that the detected metabolites overlapped well between them.

### 2.2. Analyte Recovery

In total, 244 polar metabolites of different substance classes including amino acids, free carnitine, acylcarnitines, lysophosphatidylcholines, phosphatidylcholines, and sphingolipids were tentatively annotated in the samples extracted from all studied DBS sampling materials. The comparative analysis revealed minor differences in the detected metabolome of the samples extracted from the studied DBS samplings that may be the result of both ion suppression by contaminants specific for each DBS sampling material at the stage of MS analysis and the effect of used absorbent material on analyte recovery at the sample preparation stage. 

**Figure 2:**
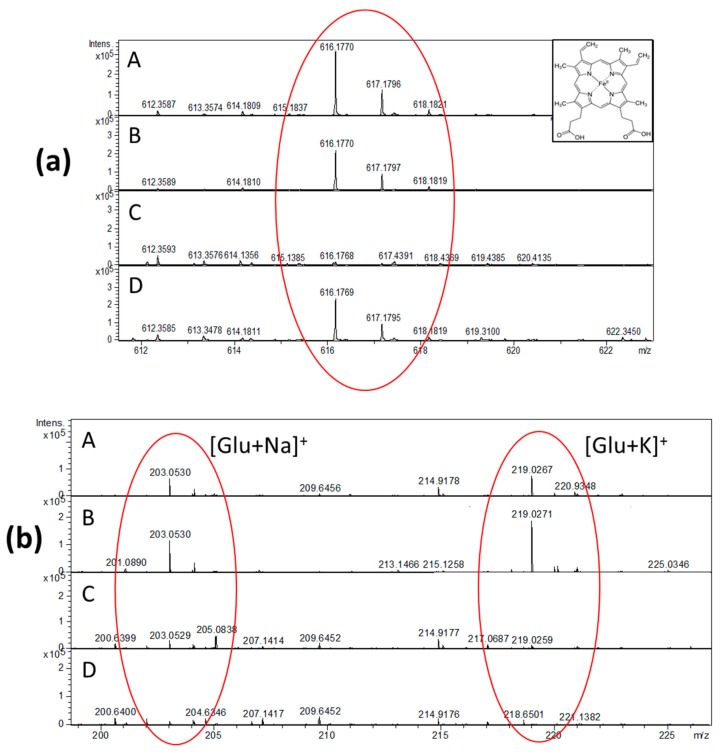
Protohaem group (C34H32N4O4Fe, *m*/*z* 616.1770) (**a**) and glucose ions (C6H12O6) with Na+ (*m*/*z* 203.0530) and K+ (*m*/*z* 219,0267) (**b**) in the MS spectra from the DBS sampling—HemaSpot™-HF Blood Collection Device (**A**); Whatman^®^ 903 Protein Saver Snap Apart Card (**B**); card ImmunoHealth™ (**C**); and glass fiber strip ImmunoHealth™ (**D**).

The analyte recovery as the effectiveness of the extraction recovery of analytes from the absorbent materials into the liquid phase significantly varied between the DBS sampling devices. In this study we used an extraction method with methanol as more affective for all metabolite classes recovery and more appropriate to obtain much cleaner extracts for DIMS [34,35,36,37]. The resulted extracts had a slight brownish color, indicating the presence of hemoglobin. Indeed, heme as a red blood cells (RBC) intracellular metabolite was extracted from all studied DBS sampling materials, however it was found in lower amount at glass fiber strip ImmunoHealth™ compared with the other DBS materials (Figure 2a). Meanwhile, glucose was better extracted from HemaSpot™-HF cartridge and card Whatman^®^ 903, while ImmunoHealth™ card and strip showed a lower recovery (Figure 2b), showing that effectiveness of extraction recovery from the used absorb material may vary from one compound to the other.

### 2.3. Metabolite Stability

Due to a variable time that might pass between the collection and the metabolomics analysis, the understanding how the storage at room temperature can affect the stability of the metabolome is of great importance. For the examination of the stability of metabolites over time the DBS samples were stored at room temperature and extracted at different time points, after seven, 14, 21, and 28 days storage.

**Figure 3:**
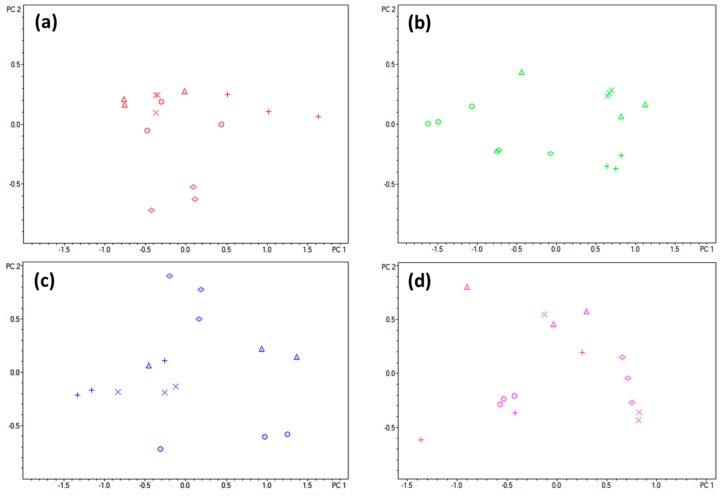
Principal component analysis (PCA) of the mass spectrometry-based metabolomics data detected from the different DBS sampling: HemaSpot™-HF Blood Collection Device (**a**), Whatman^®^ 903 Protein Saver Snap Apart Card (**b**), card ImmunoHealth™ (**c**), and glass fiber strip ImmunoHealth™ (**d**) during four weeks of storage at room temperature (zero days (o), seven days (∆), 14 days (+), 21 days (×), and 28 days (◊)) Color code: red represents samples extracted from HemaSpot™-HF Blood Collection Device; green represents samples extracted from Whatman^®^ 903 Protein Saver Snap Apart Card; blue represents samples extracted card ImmunoHealth™; and violet represents samples extracted from glass fiber strip ImmunoHealth™.

In fact, the metabolic profile changed over time when DBS samples were left at room temperature since most of the metabolites underwent degradation process overtime (Figure 3). The PCA showed that metabolite profiles of samples extracted from the DBS samplers after three to four weeks of storage were significantly differed in comparing to the extracted at first two weeks. For HemaSpot™-HF, the MS data obtained from the samples extracted at first three weeks were clustered together and separated from data from the samples extracted at last fourth week (Figure 3a). While for glass fiber strip ImmunoHealth™ the data obtained from the samples extracted at one to four weeks were clearly separated from data of the samples extracted at initial (Figure 3d). For example, for all studied DBS samplings, the PCA separation of metabolite profiling data obtained during the room temperature storage was associated with changes in peak intensity of heme and some phospholipids, but their contribution level to explaining the variances between profiles have shown that the degradation process was initiated at the different time point for different samplers. On the contrary, the contribution of low molecular weight metabolites as amino acids in the observed PCA separation was not detected, which indicates nonsignificant changes in their peaks intensity.

**Figure 4:**
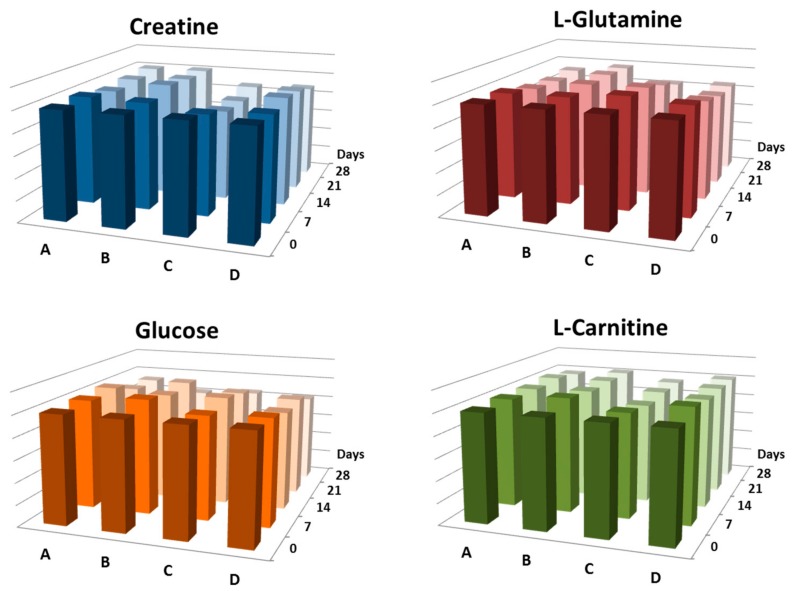
Stability of clinically relevant compounds for four weeks storage of DBS samples: HemaSpot™-HF Blood Collection Device (**A**), Whatman^®^ 903 Protein Saver Snap Apart Card (**B**), card ImmunoHealth™ (**C**), and glass fiber strip ImmunoHealth™ (**D**). The stability of metabolites from the DBS samples was determined by comparing the level of each analyte against those of the control samples (day zero). The variability of each value did not exceeded 15%.

However, it was found that the number of detected metabolites ions has not changed and for most clinically relevant compounds (creatine, l-glutamine, glucose, and l-carnitine) the differences in analytical performance are of minor incidence and they showed a slow gradual decrease in concentration during four weeks of storage (Figure 4). The molecular stability was determined by comparing the level of each analyte against those in the control samples (day zero). Nevertheless, the degradation process was not the same for all metabolites and depended on the DBS sampling material. For instance, the degradation of creatine started after seven days of storage and continued until four weeks of storage with variation rate higher for samples extracted from card ImmunoHealth™ in comparison with other studied DBS sampling materials. In contrast, the degradation of l-glutamine started after three weeks of storage and was more considerable for samples extracted from cartridge HemaSpot™-HF and strip ImmunoHealth™. The degradation trends for glucose and l-carnitine were similar to each other and were the same for all studied DBS sampling materials.

## 3. Discussion

DBS sampling is a simple and less invasive method to collect the samples for investigation and clinical analysis. Considering the development of personalized precision medicine and involving a patient in healthcare, the manufacturers produced more and more new technically upgraded devices for DBS sampling enabling to facilitate both sample collection procedure for the patient and further analysis for clinicians [22,23,24]. We here present the comparative study of four different DBS sampling devices HemaSpot™-HF Blood Collection Device, Whatman^®^ 903 Protein Saver Snap Apart Card, card ImmunoHealth™, and glass fiber strip ImmunoHealth™ in order to evaluate their compatibility for analysis of the global metabolites profile in terms of the analyte recovery and molecular stability during storage at room temperature. Our results demonstrate that all studied DBS sampling devices showed consistent analytical performance in terms of a variety of detected metabolites and no dramatic changes in the metabolic composition during four weeks of storage at room temperature. Thus, all four types of DBS sampling materials have the potential for metabolomics-based studies including the application for new diagnostic test development. 

In general, metabolomics studies can be divided into two types, depending on experimental workflow and required biological information: hypothesis-driven (targeted metabolomics) and data-driven (untargeted metabolomics) [38]. The first group of studies is usually performed to realize a well-defined hypothesis and to investigate small numbers of certain metabolites. However, for many metabolomics studies, only a general hypothesis can be developed, and, for example, some metabolome differences between healthy individuals and patients with particular diseases are supposed. In such cases, it is important to obtain reliable data on numerous metabolites present in multiple classes or metabolic pathways and to analyze large cohorts of individuals to provide statistically significant results. Therefore, data-driven investigations, especially clinical trials, cohort- and papulation-based studies requiring collection, shipment, storage, and analysis of hundreds and thousands of samples could benefit from the DBS sampling advantages [39,40]. 

Ideally, the sample preparation and mass spectrometry-based method suitable for untargeted metabolomics should be able to detect the broadest possible range of metabolites, and at the same time, it should be simple and fast to perform high-throughput analysis and to prevent metabolite loss and/or degradation. Despite the extraction, protocols are sample dependent and specific procedures are recommended for plasma/serum and DBS samples, respectively [41,42]. To evaluate the comparability of four different types of DBS sampling, we used the simple one-step extraction protocol using the methanol solution with subsequent mass spectrometry-based analysis using the direct injection mass spectrometry analysis (DIMS) as a typical workflow designed for routine untargeted analysis of the polar blood metabolome requiring limited patient material [36,37,38,43]. 

Since the analyzed samples were collected from the same person at the same time in order to exclude all possible biological variances, including hematocrit-related [27] and the studied DBS, sampling devices were produced using different absorbent materials. The variability in metabolites profiles extracted from each DBS sampling is due to effect of a specific matrix. Due to ion suppression, the specific contaminants originating from the DBS matrix and typically characterized by high ionization efficiency may influence on the ionization of analyzed compounds and thereby decrease detectable limit and resulted metabolites profile [44]. Our findings indicate that specific contaminants of each DBS sampling material effect on blood metabolome analysis (Figure 1b). However, taking this into account and applying the appropriate data processing (e.g., subtraction of material baseline) may allow to manage with this (Figure 1c). Obviously that specific contaminants and their level can be a result of particular DBS matrix’s manufacturing process, by which raw materials are transformed into material with special properties, and this process is apparently different for the studied DBS sampling materials. However, it should be noted even after specific DBS matrix contaminants subtraction, although the PCA couldn’t clearly separate the metabolomes extracted from the studied DBS samplings (Figure 1c). Some differences remained due to the effect of the matrix on the analyte recovery (Figure 2a,b). In addition, matrix effect on analyte recovery can result in decrease of method sensitivity for some metabolites [45]. In contrast to LC-MS methods DIMS is nonselective and allows detecting several hundred metabolites of different substance classes in one run. In the samples extracted from all studied DBS sampling materials, we tentatively annotated 244 polar metabolites including amino acids, free carnitine, acylcarnitines, lysophosphatidylcholines, phosphatidylcholines, and sphingolipids. Thus, all four DBS sampling devices enable to analyze most of the endogenous metabolites, which are covering metabolic pathways with central biological relevance that expand the potential clinical application. Moreover, the analysis of whole blood metabolome extracted from DBS samples of capillary blood enables to detect the extra metabolites which are not presented in plasma/serum. For instance, in the extracted metabolome, we found an RBC intracellular metabolite heme, which is typically present in high amount in the RBC metabolome (Figure 2a).

The advantages of DBS include the possibilities for easy shipment and storage without the maintenance of specific temperature. Depending on where the patient is at the time of sampling (at home or at clinic) and how long it will take to deliver the sample to the laboratory a variable time frame (from several hours to several weeks) might pass between the collection and the metabolomics analysis. Therefore, to evaluate the use of various DBS sampling methods for metabolomics applications, especially for clinical diagnostics, it is important to check the stability of the samples at room temperature storage for this time period. Our results have shown that molecular stability of DBS samples is analyte and matrix-dependent with enhanced degradation trend over time of storage for majority of metabolites, which is consistent with previous studies (Figure 3) [18,46,47]. However, the number of detected metabolite ions has not changed, and the rate of the degradation process is not the same for all analytes and for different metabolites the degradation started after different time of storage. For instance, the peaks of heme and some phospholipids were significantly reduced in intensity during the storage time due to their susceptibility to oxidative damage [46,48]. Therefore, the decrease of the storage temperature is recommended to enhance metabolites stability, especially for long-term studies, but it is clear that the need to transport samples at low temperature may nullify the main advantage of DBS. Our study demonstrated that changes in intensity levels of metabolites with high contribution to the samples separation were occurred at the different time point for different samplers. In a case of glass fiber strip ImmunoHealth™, these changes were observed even in a week of storage, whilst of other DBS samplings the considerable discrimination of samples was observed only after three weeks. It would seem the mechanism of metabolite degradation with time and storage temperature including oxidation or hydrolysis and the action of blood/plasma enzymes should be the same for all DBS materials, but our findings could be the result of irreversible adsorption or specific chemical reactions of each DBS sampling material. Nevertheless, for most clinically relevant compounds (creatine, l-glutamine, glucose, and l-carnitine) the differences in analytical performance were of minor incidence and they showed a slow gradual decrease in concentration during four weeks of storage (Figure 4), allowing the analysis to be carried out at a considerable time after collection in some cases. In other cases, the DBS storage should be at temperature ≤ 20 °C [46,47,48]. Although in 2017, the researchers from Pacific Northwest National Laboratory (USA) have shown that lipid quantitation in the 15-year-old DBS samples stored at room temperature is possible [49].

In summary, we demonstrated the compatibility of four different DBS sampling devices for metabolomics analysis in terms of their analyte recovery and stability for blood metabolite profiling. Main differences were observed for the effectiveness of extraction recovery of analytes from card and glass fiber strip produced by ImmunoHealth™ that could be the result of both absorbent material effect at the sample preparation stage and ion suppression due to specific interfering contaminants at the MS analysis stage. However, if these factors are taking into account investigation design, any studied DBS sampling may be used with application of appropriate algorithm for further analysis (e.g., baseline subtraction for contaminants MS peaks removing, use of internal standard to account of ion suppression effect). Although all mentioned advantages of using DBS samples before traditional plasma/serum samples are obvious, it should be noted that the quality of DBS samples can be poor because patients often do not follow instructions concerning sampling, drying, and mailing. Therefore, proper validation and regulatory approval for new methods of DBS sample collection, transport, and further clinical analysis are required.

## 4. Materials and Methods 

### 4.1. DBS Sampling Materials

**Figure 5:**
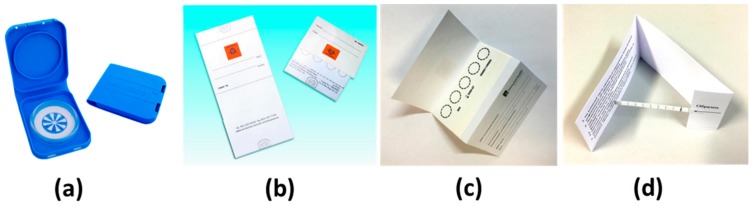
DBS sampling—HemaSpot™-HF Blood Collection Device (**a**); Whatman^®^ 903 Protein Saver Snap Apart Card (**b**), card ImmunoHealth™ (**c**), and glass fiber strip ImmunoHealth™ (**d**).

The HemaSpot™-HF Blood Collection Device was purchased from Spot On Sciences, (San Francisco, CA, USA). HemaSpot™-HF is a cartridge containing an absorbent paper and desiccant covered with an application surface that contains a small opening to allow entry for blood (Figure 5a). The Whatman 903^®^ Protein saver snap apart cards (GE Healthcare) were purchased from Merck KGaA (Darmstadt, Germany). The 903 paper in this device, imprinted with four half-inch circles, is enclosed between two pieces of cover stock (Figure 5b). The card ImmunoHealth™ is an analog of Whatman^®^ 903 paper, imprinted with five half-inch circles, and was supplied by Immunoved (Moscow, Russia) (Figure 5c). The glass fiber strip ImmunoHealth™, 0.5 cm and 1 cm-width sampling strips, were fabricated from glass fiber membrane (MAPDS-0300, Arista Biologicals, Allentown, PA, USA) and supplied by Immunoved (Moscow, Russia). 0.5 cm width membrane was supplied with black marks at every 0.5 cm. (Figure 5d) 

### 4.2. Samples

For the DBS matrix study, all the samples were collected from the same person (male) at the same time in order to exclude all possible deviations deriving from biological variances between patients including hematocrit effect. After finger pricks using a single-use sterile safety lancet, the capillary blood drops were collected by capillary tube and the same blood volume (20 μL) was directly pipetted onto HemaSpot™-HF cartridge, paper cards Whatman^®^ 903 and ImmunoHealth™, and glass fiber strip ImmunoHealth™ sequentially and as per manufacturer’s instructions. It was ensured that the blood filled the all marked absorbent material uniformly. After the samples were air dried completely for 3 h at room temperature (~20–22 °C), they were stored at room temperature in zip-closure foil bags (GE Healthcare, Westborough, MA, USA). For the DBS storage stability study, the samples stored for four weeks. Diets and other physiological parameters were not controlled in this study. The participant gave his written informed consent and the study was approved by the local ethical authorities.

### 4.3. Metabolomics-Based Samples Preparation

For further analysis of low molecular weight fraction (metabolome) of blood, the filled segments from DBS samples were cut out and placed in clear Eppendorf Safe-Lock 1.5 mL Tubes (Eppendorf AG, Hamburg, Germany). To remove proteins and extract metabolites, 40 µL of water (Sigma-Aldrich, St. Louis, MO, USA) and 160 µL of methanol (J.T.Baker, Gliwice, Poland) were added to each DBS sample, mixed and incubated at room temperature during 20 min. After incubation samples were centrifuged at 13,000× *g* (Centrifuge 5804R; Eppendorf AG, Hamburg, Germany) for 15 min at room temperature, and resultant supernatants were then transferred to clean Eppendorf tubes and centrifuged one more time. Before mass spectrometry analysis, each sample was diluted 50-fold with methanol with 0.1% formic acid (Sigma-Aldrich, St. Louis, MO, USA) and the internal standard (IS) Losartan (C22H23ClN6O, *m*/*z* = 423.169) was added to an end concentration of 10 ng/mL to obtain the analyzed solution. To determine the baseline effects of the DBS matrix, clean and empty segments (blank) were cut out and processed in parallel to sample segments. For the examination of the stability of metabolites over time, the DBS samples were analyzed after one week, two, three, and four weeks storage at room temperature. All chemicals and solvents were of HPLC and UHPLC grade.

### 4.4. MS Analysis

Mass spectrometry analysis of the samples was carried out by direct injection to a hybrid quadrupole time-of-flight mass spectrometer (maXis Impact, Bruker Daltonics, Bremen, Germany) equipped with an electrospray ionization (ESI) source. The mass spectrometer was set up to detect ions with the mass-to-charge ratio (*m*/*z*) in the range from 50 to 1000 Da and mass accuracy up to three parts per million (ppm). The appropriate mass range of the mass spectrometer was calibrated by using ES Tuning Mix (Agilent Technologies, Santa Clara, CA, USA). Spectra were acquired in the positive ion mode detection. The samples were injected into the ESI source using a glass syringe (Hamilton Bonaduz AG, Bonaduz, Switzerland) and a syringe injection pump (KD Scientific Inc., Holliston, MA, USA) with flow rate of 180 µL/h for one minute. All samples were analyzed in random order and in three-five technical replicates. MS/MS spectra of selected precursor ions with the intensity threshold of 5000 were acquired at collision energy from 10 to 40 eV.

### 4.5. Data Processing and Analysis

Mass spectrometry raw files were analyzed in DataAnalysis software (version 3.4, Bruker Daltonics, Bremen, Germany) by summarizing signals for one minute for recalibration, peak detection, peak intensity, and area calculation. Ion metabolite masses were determined from the mass spectrum peaks selected with the following parameters: peak width – 5, signal to noise ratio – 2, and relative and absolute threshold intensity – 0.05% and 100, respectively. Alignment of mass spectrum peaks, removal of low-informative peaks, and data correction to address ionic inconsistency in blood plasma samples were performed by using the self-made algorithm in Excel. It was considered that two peaks relate to the same metabolite ion if their mass difference does not exceed 0.01 Da. Peaks that were detected in <80% of technical replicates of each DBS sampling and each time point were removed from the analysis. All peaks intensities and area values were normalized by the internal standard (IS) Losartan (C22H23ClN6O, *m*/*z* = 423.169) concentration levels. The results are expressed as mean ± standard deviation (SD). Statistical significance was calculated by the Kruskal-Wallis test. Principal Component Analysis (PCA) to assess differences among the compared DBS sampling materials was performed on the acquired mass spectrometry metabolite profiling data using the ProfileAnalysis software (version 2.1, Bruker Daltonics, Bremen, Germany). 

The selected features were tentatively annotated by using accurate molecular weight and MS/MS information and via database searching such as the Human Metabolome Database (HMDB) [50] and METLIN [51]. The identification of species was based on the molecular weight, at least one specific fragment, and structure (see Figures S1–S4).

## 5. Conclusions

Our results indicate that the analytical performance of all tested DBS samplings showed consistent results overall detected metabolites and no distinguished changes between them in the metabolic composition during the four weeks of storage at room temperature. Based on our data it is recommended to select an appropriate DBS sample collection device depending on the design of the study. In addition, the described methodology using different DBS sampling demonstrates the potential for enabling patients to contribute to the expanding bioanalytical demands of precision medicine and population health studies.

## Supplementary Materials

The following are available online at https://www.mdpi.com/2218-1989/9/11/277/s1, Figure S1: MS/MS spectrum with specific fragments for glucose, Figure S2: MS/MS spectrum with specific fragments for creatine, Figure S3: MS/MS spectrum with specific fragments for L-glutamine, Figure S4: MS/MS spectrum with specific fragments for L-carnitine.
