# Supplementary figures and images for: Evaluation of Dried Blood Spot Sampling for Clinical Metabolomics: Effects of Different Papers and Sample Storage Stability
Source: Metabolites. 2019 Nov 12;9(11):277. doi: 10.3390/metabo9110277 (PMC6918358; doi:10.3390/metabo9110277)

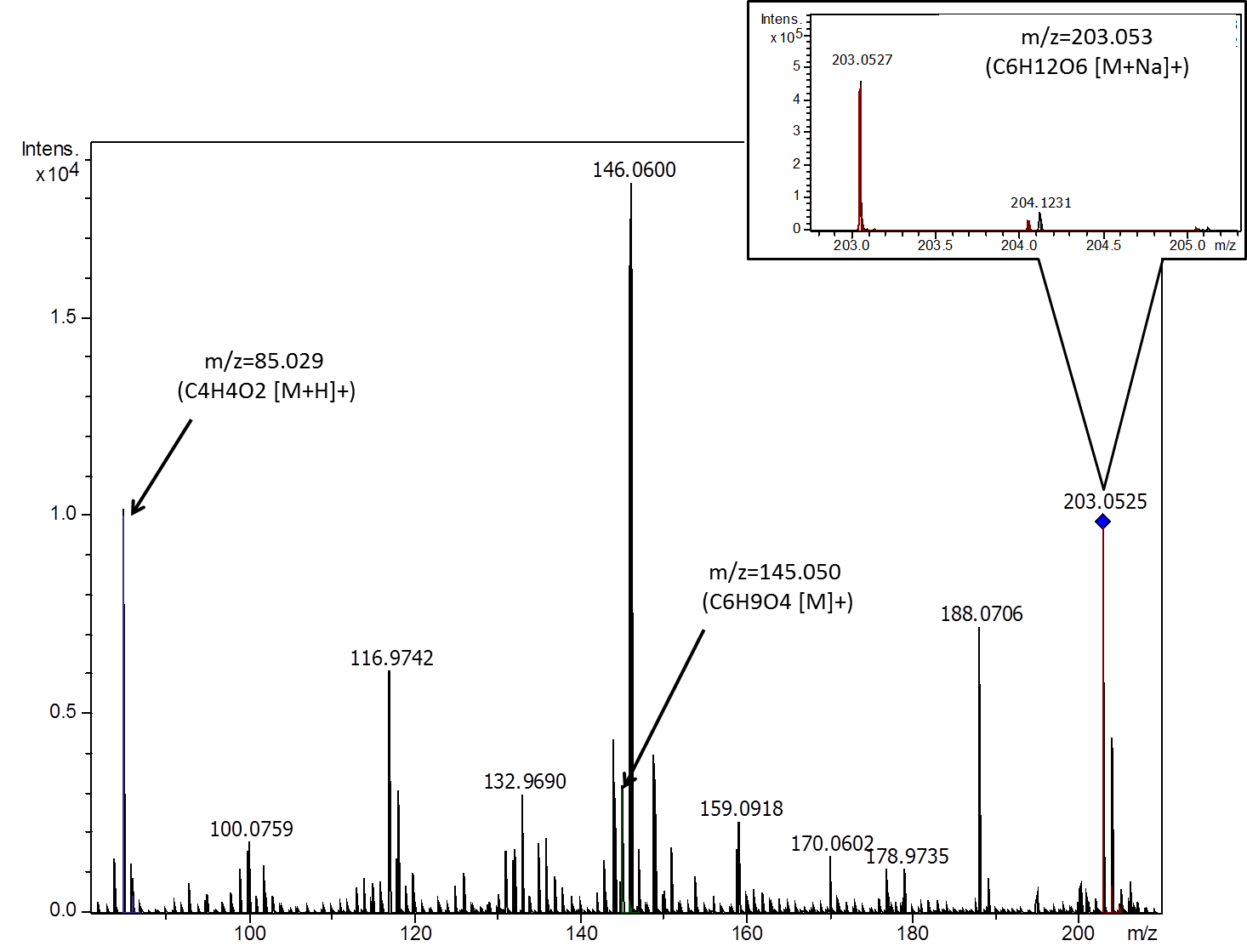

Supplement: Supplementary file 1 [file metabolites-09-00277-s001.zip › Supplementary Materials/Figure S1.tif]

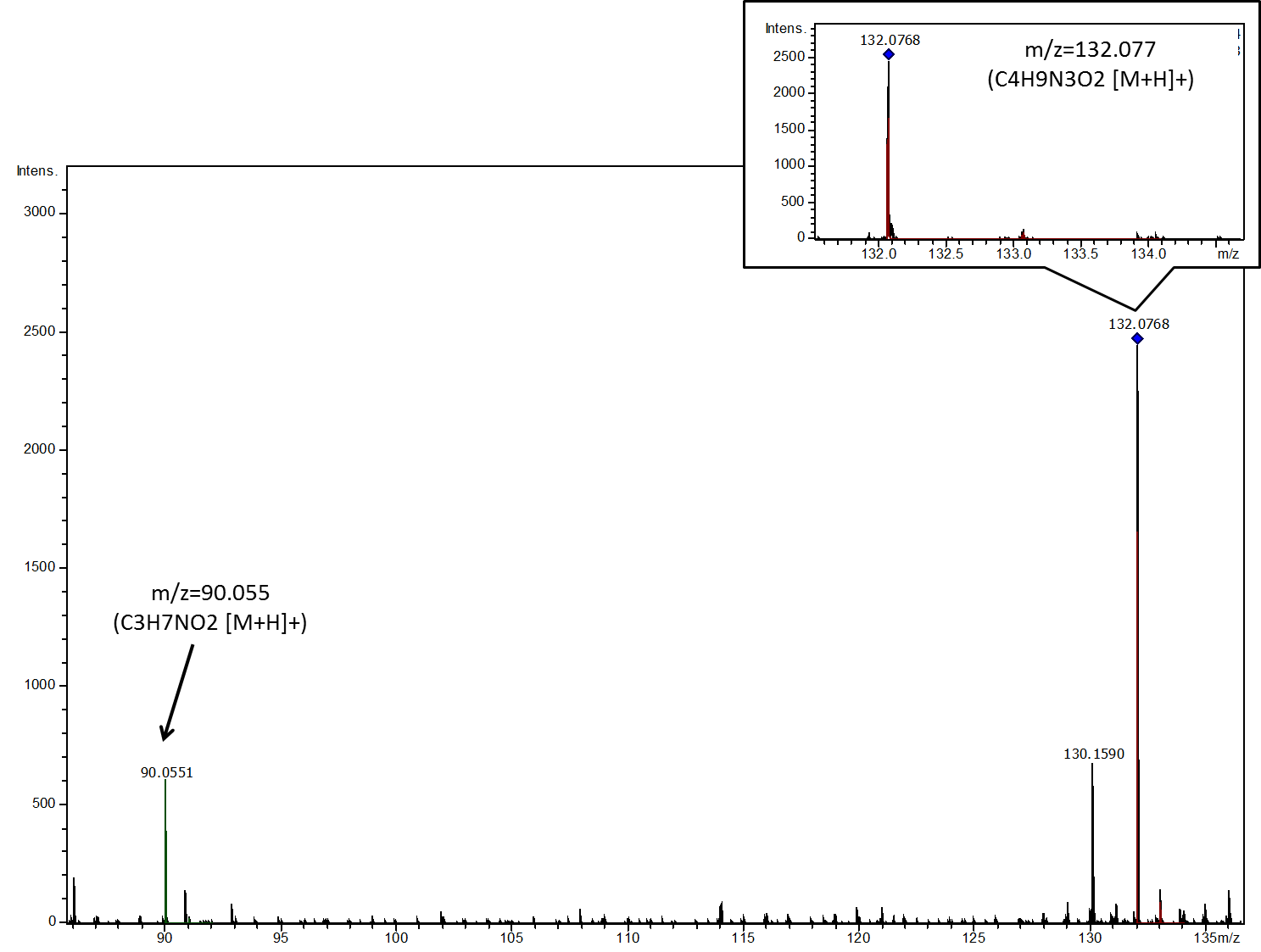

Supplement: Supplementary file 1 [file metabolites-09-00277-s001.zip › Supplementary Materials/Figure S2.tif]

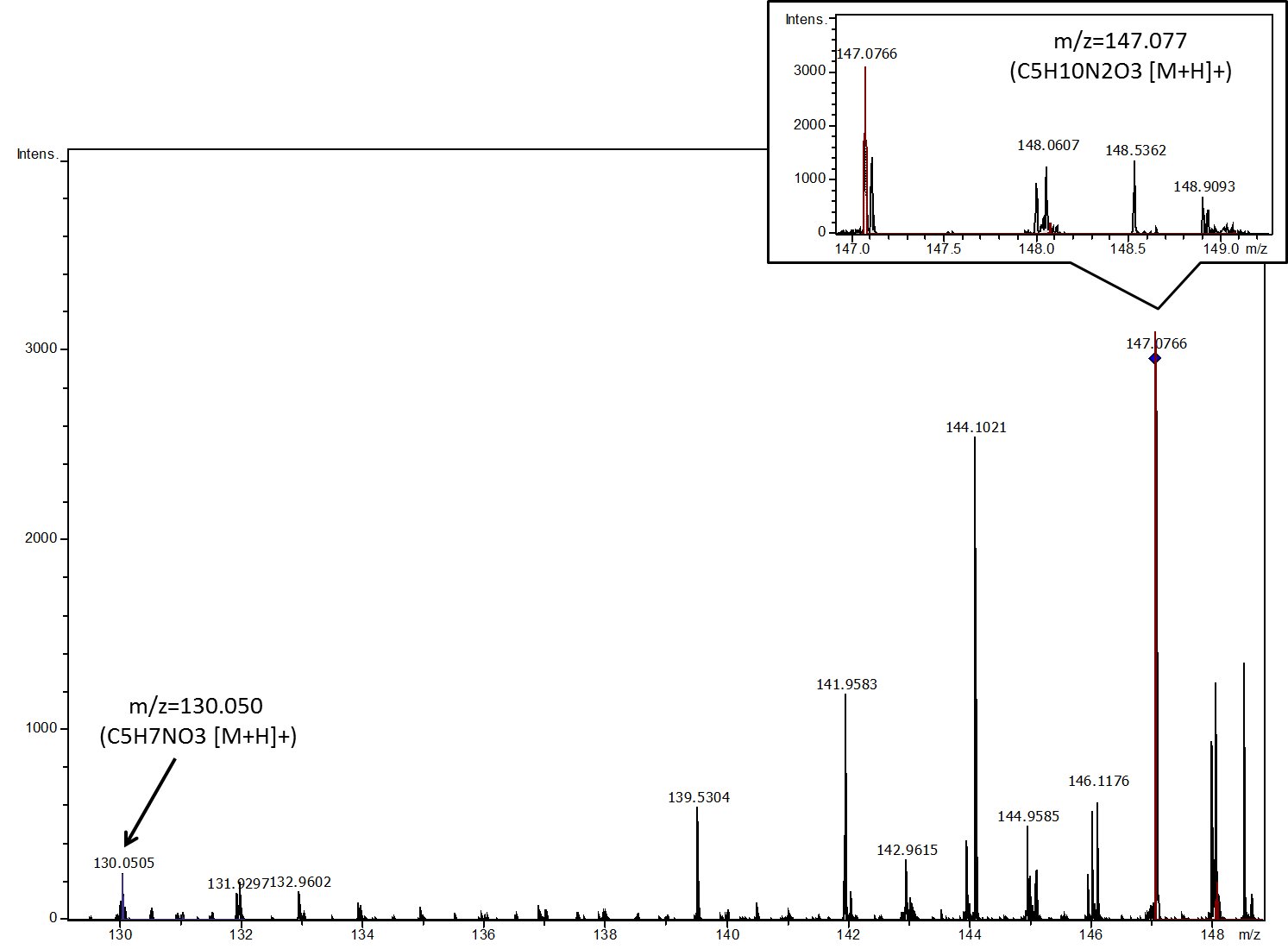

Supplement: Supplementary file 1 [file metabolites-09-00277-s001.zip › Supplementary Materials/Figure S3.tif]

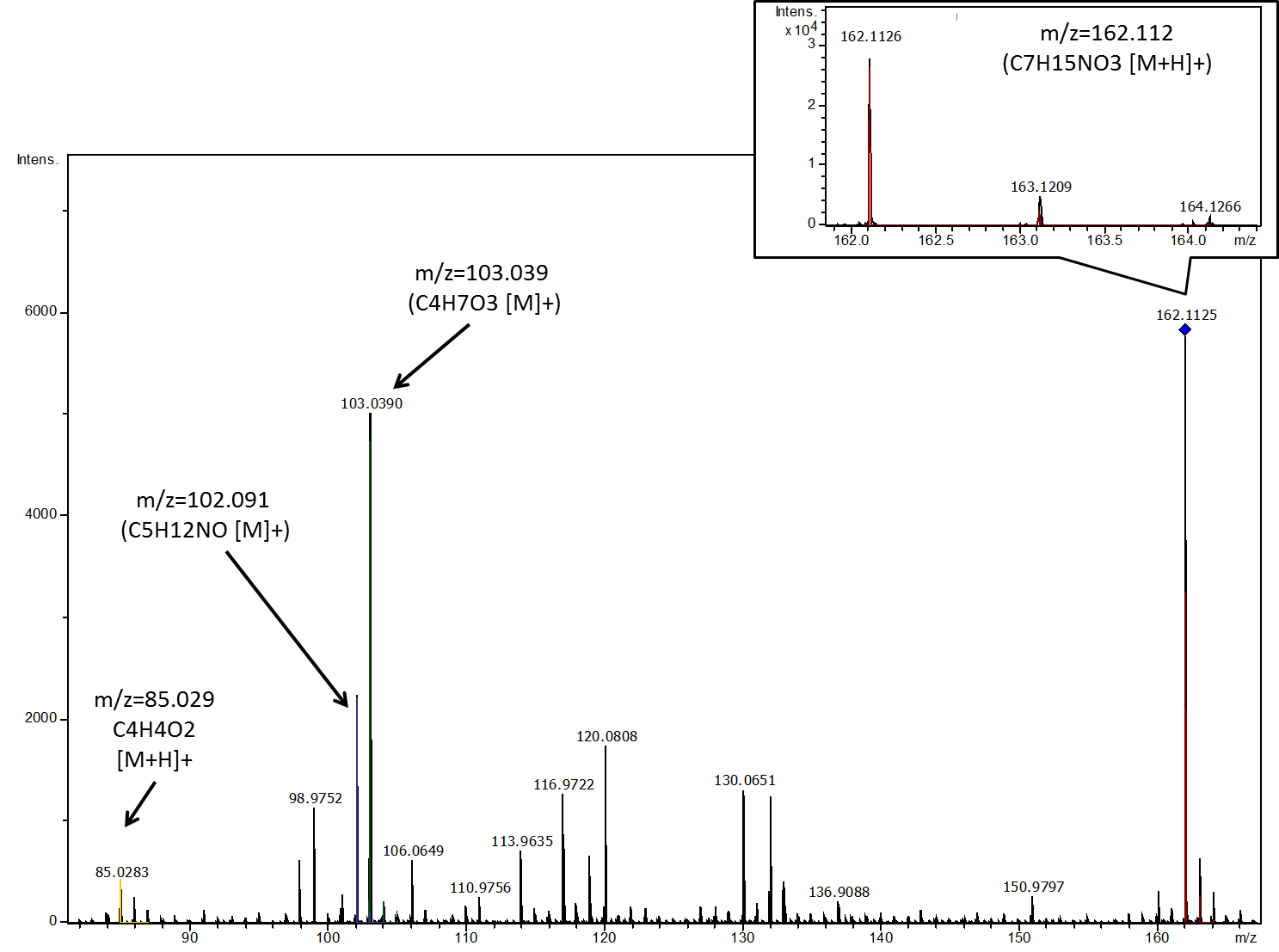

Supplement: Supplementary file 1 [file metabolites-09-00277-s001.zip › Supplementary Materials/Figure S4.tif]
